# Gaps in universal health coverage in Malawi: A qualitative study in rural communities

**DOI:** 10.1186/1472-6963-14-234

**Published:** 2014-05-22

**Authors:** Gilbert Abotisem Abiiro, Grace Bongololo Mbera, Manuela De Allegri

**Affiliations:** 1 Institute of Public Health, Medical Faculty, University of Heidelberg, Heidelberg, Germany; 2 Department of Planning and Management, Faculty of Planning and Land Management, University for Development Studies, Wa, Ghana; 3 Research for Equity and Community Health Trust (REACH Trust), Lilongwe, Malawi

**Keywords:** Universal health coverage, Financial protection, Access to health care, Gaps in coverage, Geographical inequities, Community perspective, Qualitative study, Malawi

## Abstract

**Background:**

In sub-Saharan Africa, universal health coverage (UHC) reforms have often adopted a technocratic top-down approach, with little attention being paid to the rural communities’ perspective in identifying context specific gaps to inform the design of such reforms. This approach might shape reforms that are not sufficiently responsive to local needs. Our study explored how rural communities experience and define gaps in universal health coverage in Malawi, a country which endorses free access to an Essential Health Package (EHP) as a means towards universal health coverage.

**Methods:**

We conducted a qualitative cross-sectional study in six rural communities in Malawi. Data was collected from 12 Focus Group Discussions with community residents and triangulated with 8 key informant interviews with health care providers. All respondents were selected through stratified purposive sampling. The material was tape-recorded, fully transcribed, and coded by three independent researchers.

**Results:**

The results showed that the EHP has created a universal sense of entitlements to free health care at the point of use. However, respondents reported uneven distribution of health facilities and poor implementation of public-private service level agreements, which have led to geographical inequities in population coverage and financial protection. Most respondents reported affordability of medical costs at private facilities and transport costs as the main barriers to universal financial protection. From the perspective of rural Malawians, gaps in financial protection are mainly triggered by supply-side access-related barriers in the public health sector such as: shortages of medicines, emergency services, shortage of health personnel and facilities, poor health workers’ attitudes, distance and transportation difficulties, and perceived poor quality of health services.

**Conclusions:**

Moving towards UHC in Malawi, therefore, implies the introduction of appropriate interventions to fill the financial protection gaps in the private sector and the access-related gaps in the public sector and/or an effective public-private partnership that completely integrates both sectors. Current universal health coverage reforms need to address context specific gaps and be carefully crafted to avoid creating a sense of universal entitlements in principle, which may not be effectively received by beneficiaries due to contextual and operational bottlenecks.

## Background

Many low- and middle-income countries (LMICs) have embarked on health system reforms aimed at achieving Universal Health Coverage (UHC) [1,2]. Such reforms are designed to introduce or expand public health care financing systems to pool resources across a wide range of prepaid financing sources, as replacements for out-of-pocket payments [2-4]. The policy objective of universal health coverage is to ensure that all residents of a nation (universal population coverage) enjoy adequate coverage by prepaid financing systems (universal financial protection) and have access to needed health services of good quality (universal access) [2,4,5].

These three main dimensions of universal health coverage: population coverage, financial protection and access to services, are inter-linked and interdependent [4]. Universal population coverage is attained when there is no systemic exclusion of certain population groups (especially the poor and vulnerable) and when all residents enjoy the same entitlements to the benefits of public funding, irrespective of their political affiliations, nationality, race, gender, socio-economic status or geographic locations [2,3,6-9]. Universal financial protection is attained in the absence of: (substantial) out-of-pocket payments; fear of and delay in seeking healthcare due to financial reasons; borrowing and sales of valuable assets to pay for healthcare; and critical income losses due to health care payments [2,6]. Universal access includes a number of sub-dimensions: availability of health services, personnel and facilities; accessibility of health services based on users’ location relative to health services and transportation possibilities; acceptability in terms of appropriate client-provider relationships and attitudes towards each other; accommodation in terms of timeliness, appropriateness and quality of services; and affordability in terms of cost of services relative to clients ability-to-pay [5,8,10-13]. UHC can only be realized if universal access is attained in conjunction with a realization of the other two dimensions of UHC such as universal population coverage and financial risk protection. A deficiency in any aspect of these three main dimensions signifies a gap that needs to be filled for UHC to be achieved.

Global debates [5,7,14], and to some extent national level aggregates and economic modeling [15-18], have extensively been used to ascertain gaps in universal health coverage in various contexts and to postulate possible solutions. Less attention has been paid to the identification of context specific gaps in universal coverage from the perspective of the community. This paucity of evidence is somewhat disturbing considering that the World Health Organization recognizes responsiveness as an intrinsic objective of any health care system [19], one that needs to be maintained in the quest towards universal health coverage [20]. This underscores the fact that reforms have often been implemented following a top-down approach, with little attention being paid to documenting and exploring gaps in coverage as experienced by communities [21].

This qualitative study aims to fill this knowledge gap by exploring how rural communities in sub-Saharan Africa (SSA), specifically in Malawi, experience and define gaps in the coverage provided by their health care system. The rationale is to ensure that future interventions, within this context, are aligned with people’s actual needs; respecting responsiveness as an explicitly acknowledged intrinsic policy objective of UHC reforms [20].

Malawi is a low-income sub-Saharan African country with a population of approximately 15 million people [22]. The majority (80%) of the population live in rural areas and depend on rain-fed agriculture for their livelihood [23]. The Gross Domestic Product (GDP) per capita (purchasing power parity (PPP) in 2012) is approximately 900 United States Dollars (USD) [24]. About 60% of the population live below the international poverty line of 1.25 USD a day [22].

The average total healthcare expenditure of Malawi stands at about 34 USD per capita, equivalent to 12.3% of GDP [25]. The proportion of government expenditure on health is 2.1% of GDP and this constitutes about 18.0% of total healthcare expenditure [25]. Health service provision relies on a public-private mix of providers. Over 60% of all health services are provided in public hospitals and health centers, 37% by the private not-for-profit Christian Health Association of Malawi (CHAM) and the rest by individual private-for-profit health practitioners [23].

Since 2004, full-cost coverage of an Essential Health Package (EHP) has been implemented in Malawi as a step towards UHC. The EHP includes about 55 interventions which reflect the main morbidity and mortality patterns of the country (see Table 1) [25,26]. The EHP is funded from general tax revenue and donor funds. It is supposed to be provided free of charge in all public facilities, and at the selected CHAM facilities bound by Service Level Agreements (SLAs) with the government [25]. Only a few employers and the Medical Aid Society of Malawi (MASM) offer private health insurance to formal sector employees [27]. The rest of the population has no access to complementary health insurance [28]. A number of studies have quantified inequities in access and health outcomes, suggesting the existence of important gaps in coverage [26,29-36]. A recent quantitative analysis identified remarkable weaknesses in actual EHP provision and attributed it to problems of under-funding [26].

**Table 1:** Broad components of the Malawi Essential Health Package [25,26]

***Package***	***Broad categories of services***
*Initial designed Package*	*● Prevention and treatment of vaccine-preventable diseases*
	*● Management of acute respiratory infections (ARI) including pneumonia.*
	*● Malaria prevention and treatment i.e. using insect treated nets (ITNs) and active case management.*
	*● Reproductive health interventions to address adverse maternal/neonatal outcomes (family planning, maternal and neonatal health, PMTCT)*
	*● Prevention and control of tuberculosis*
	*● Prevention and treatment of acute diarrhoea diseases including cholera*
	*● Prevention and treatment of HIV/AIDS and other sexually transmitted infections (STIs)*
	*● Prevention and treatment of schistosomiasis*
	*● Prevention and treatment of malnutrition and nutritional deficiencies.*
	*● Prevention and management of common eye, ear and skin conditions*
	*● Treatment of common injuries and emergencies.*
*Later inclusions*	*● Cancer treatment*
	*● Other non-communicable diseases*

## Methods

### Study setting, design, sampling and data collection

We conducted our study in Thyolo and Chiradzulu, two rural districts in Southern Malawi, with a combined population of 878,401, equivalent to 6.7% of the country’s population [37]. The districts have about 54 health facilities, comprised of 37 public, 13 CHAM and 4 private-for-profit facilities.

The data was collected from August to September 2012, within the framework of an exploratory qualitative study aimed at informing a subsequent discrete choice experiment [28]. Within the context of this study, we purposely collected information on perceived and experienced gaps in universal health coverage from the target population.

We collected information from both adult community residents and health workers from selected health facilities. Sampling and recruitment procedures have been described in detail elsewhere [28]. In brief, based on an anticipated saturation point, stratified purposive sampling was used to select participants for 12 focus group discussions (FGDs) (size = 8-12 participants each) among community residents and 8 key informant interviews with health workers. A total of 127 community residents, distributed in 6 rural communities, participated in the 12 FGDs (6 with men and 6 with women). An equal number of FGDs and key informant interviews were completed in the two districts. Because of their experiences with the challenges that community residents face when seeking care, health workers were included in the study as key informants to enhance the credibility of the findings, by cross-checking their responses with the answers provided by community residents [38]. The health workers were selected from purposefully sampled healthcare facilities to reflect variations in healthcare provision in the study area. The sampled facilities were comprised of: two public district hospitals (Thyolo and Chiradzulu district hospitals); two public health centers (Chivu in Thyolo and Ndunde in Chiradzulu); two private-not for-profit (CHAM) hospitals (Adventist –Malamolo in Thyolo and St. Joseph -Nguludi in Chiradzulu); and two private-for–profit clinics (Hiwa in Thyolo and Akasale in Chiradzulu). The health workers that were interviewed from these facilities were comprised of: two medical doctors, two nurses/midwives, two medical assistants, one clinical officer and a paramedic. All study participants were identified, contacted, and recruited with the help of community leaders and trained research assistants.

All FGDs and key informant interviews were conducted in secure enclosed places at the community and facility levels, respectively. Due to the less sensitive nature of the study topic, FGDs made it relatively easier to explore consensus and differences in opinions on UHC gaps among community residents. To respect local socio-cultural concerns, FGDs were gender-specific and included either only men or only women. All FGDs were conducted in the local language (Chichewa) by a trained facilitator, accompanied by a note-taker. The first author conducted all interviews with health workers in English. Two different, but mirrored, interview guides containing open-ended questions and probes were used to facilitate both the FGDs and the key informant interviews. The relevant sections of the guides covered the following topics: cost and payments associated with seeking health care, access to health providers, facilities and medical products, transportation and distance to facilities, perceived quality of health care (waiting times, perceived quality of drugs) and attitude of health workers, among others (see Additional file 1). Prior to field work, both guides were pre-tested and modified to reflect the pretest experience. All FGDs and key informant interviews were tape-recorded and transcribed verbatim, with the FGDs being translated into English for analysis.

### Ethical considerations

Ethical approval for the study was obtained from the Ethical Committee of the Faculty of Medicine of the University of Heidelberg and from the National Health Science Research Committee (NHSRC) in Malawi. Written informed consent was obtained from all participants prior to the beginning of the FGD/interview process. All sampled respondents consented to and participated in the study. To ensure confidentiality, respondents in the FGDs were discouraged from discussing each other’s views outside the FGD setting. Also, to make it less possible for respondents’ views to be easily linked to their personal identities, we did not record the names of the respondents. The RATS guidelines for reporting qualitative research, modified for BioMed Central instructions to authors, have been adhered to.

### Data analysis

Thematic analysis was done to identify the community’s perception of gaps in universal health coverage [38]. Analysis began with an independent reading, coding, and categorizing themes of the transcripts by all three authors. The first author coded the entire material using the NVivo 9 software. The second and last authors manually analyzed two-thirds of the material. All analysts approached the material inductively, letting codes and categories emerge as they worked through the transcripts [38]. At a later stage, the three analysts brought together the results of their analyses to identify overarching themes. Codes were re-categorized into broad and sub-themes, reflecting the various dimensions of universal coverage and the context specific issues raised in the data, respectively. Discrepancies in interpretations across the three authors were reconciled by returning to the text and to notes taken during data collection for further analysis. Findings are presented along the three-dimensional UHC framework: universal population coverage; financial protection; and access to health care. To avoid redundancy, affordability as a dimension of access has been reported under financial protection. Poignantly chosen quotations from the qualitative transcripts have been included in the results section to illustrate our key findings, in order to give a voice to our respondents.

## Results

### Gaps in population coverage

The FGDs with community residents did not reveal systemic exclusion of population groups on the basis of socio-economic status in the coverage of public tax funding. Community residents unanimously reported a sense of entitlement to free provision of the EHP at public facilities.

“Where we live the hospitals that we go are free for everyone, when you are admitted and when you are treated you just leave without paying anything” (FGD05: Female)

Further analysis of the FGDs, however, revealed geographical exclusion of residents from certain rural communities from effective EHP coverage. In communities where only private or CHAM facilities are located, FGD participants argued that it is practically not possible to access the EHP free of charge, since services offered by such facilities are paid on an out-of-pocket basis.

“When you go to Adventist (CHAM hospital), you pay first to see a doctor and then if you want to test, before they do the test, you pay…you pay for everything” (FGD05: Female).

Health workers in CHAM and private facilities confirmed that geographical disparities in population coverage result from the operational ineffectiveness of the SLAs. None of the private for-profit health facilities identified in the study area was under the SLA. Health workers interviewed at private-for-profit facilities reported either failing to meet the criteria for an SLA or being afraid that accepting an SLA may raise expectations among their clients that all services should be provided free of charge. Similarly, due to irregular reimbursement by the government, health workers in the sampled CHAM facilities reported providing only maternal and neonatal services under an SLA.

“It is only maternity side whereby we have a service level agreement with the government …. In the past, we used to have services agreement on pediatrics…but that service level agreement was cut off because they (government) were not paying us regularly” (Nurse/mid-wife, CHAM hospital).

### Gaps in financial protection

#### Out-of-pocket medical expenditure

All community residents who participated in the FGDs reported being charged no formal or informal fees for the treatment received at public facilities. However, they consistently reported incurring substantial out-of-pocket payments for medical treatment at CHAM/private health facilities and/or when purchasing drugs at private pharmacies. Despite their awareness of and experience with free healthcare provision at public facilities, respondents reported frequently being compelled by circumstances to seek care at CHAM/private facilities and thus, incur substantial out-of-pocket payments. They justified their need to do so in regards to a number of shortcomings in public health service provision, namely: shortages of medicines and health workers, insufficient health facilities and equipment, poor access to emergency services, long distance and transportation difficulties, poor attitude of health workers, overcrowding and perceived poor quality of care, among others.

“The government hospital … can be overcrowded and without drugs, so if other people help you with money, you go to private hospital” (FGD08: Male)

Both community respondents and health care providers consistently reported high expected out-of-pocket payments to be the main barrier to seeking care at CHAM/private facilities. As a consequence of financial unaffordability, community respondents reported delays in seeking care, refusing hospital admissions, demanding early discharge or being detained in the hospitals for non-payment of bills.

“There are times when the doctor (at CHAM) tells you to be admitted, but due to shortage of money in your pocket you can’t allow that, because admission is a bit expensive” (FGD10: Male)

“People are not able to pay for all the services so patients are discharged early because they don’t have enough money. They may require staying in the hospital for 7 days……but they stay in the hospital for three days…they are asking to be discharged just because they can’t pay for that” (Medical Doctor, CHAM).

To meet the cost of seeking the much needed care at private/CHAM facilities, community residents reported reliance on sales of farm produce, borrowing, and contributions from family members. These are all implicit indicators of gaps in financial protection.

“Relatives contribute or we borrow from friends to pay at private facilities” (FGM08: Male)

Four out of the eight health workers interviewed had additional private health insurance coverage through the Medical Aid Society of Malawi (MASM). This enabled them to access services at no direct cost, even from private/CHAM facilities that normally charge fees. At the time of the study, none of the FGD participants had any functional additional health insurance coverage, whether private or public. Similarly, only a few of the FGD participants had ever even heard of the concept of health insurance.

#### Travel expenditure

Irrespective of where care is sought, community residents reported transport as an important additional burden. Transport costs challenging residents’ ability to pay included: public transport from the patient’s home to distant health care facilities; public transport from the community level facility to the district hospital, in case of referral; and at times, calling or fueling a government-owned ambulance, in case of referral. The latter was perceived as an unexpected source of exposure to financial risk, given that emergency transport is supposed to be provided free of charge. All health workers confirmed the communities’ views and reported additional difficulties due to insufficient availability of public ambulances.

“These days, they (health workers) tell us that the patient should get his/her own transport to the district hospital, always they say they do not have fuel (for public ambulances)…….so a poor lady like me where will I get the money at that time” (FGD12: Female)

“Whenever there is a patient, we do call for an ambulance from the district but it doesn’t come in time. (so) I just ask the patient, if they can manage to go using the public transport” (Medical Assistant, public health center)

As the direct consequence of high transport costs, some community residents reported often deliberately foregoing or delaying seeking care.

“I was injured and went to…the health center where the doctors (medical assistant) referred me to Thyolo (district hospital), but I had no access to transport. As a result, I went home hunting for money and after two days that I was able to raise money for transport but it was too late and I had several complications at Thyolo” (district hospital) (FGD01: Female)

### Gaps in universal access

#### Shortcomings in public health service provision

The gaps in public provision which expose community residents to financial risk, by compelling them to seek care at private facilities, obviously represent the main gaps when considering the access dimension of universal health coverage. These gaps - shortages of medicines and health workers, insufficient health facilities and equipment, overcrowding, poor access to emergency services, long distance and transportation difficulties, poor attitude of health workers, and hence perceived poor quality of care - are further explored under the following thematic topics.

#### Availability and accessibility of health services

Frequent drug stock outs dominated the discussions in 8 out of the 12 FGDs and in all the interviews with health workers in public facilities. FGD participants suspected that drugs were being badly managed and/or purposely redirected towards private provision by the same providers serving at the public facilities. Health workers in public health facilities, however, attributed the shortages to inadequate supplies from the national drug provision system, which is heavily dependent on external donors.

“The problem is that they (health workers) are selling these government drugs to owners of the groceries around” (FGD06: Male)

“In the past, I think things were better but now things have really deteriorated. As we speak now, this month, we only got half of the medical supplies that we require per month.” (Medical doctor, public hospital)

Public sector health workers indicated that they cope with drug shortages by postponing treatment, by referring to a private pharmacy, and/or by referring clients to another health facility. Yet again, this confirms how gaps in public provision feed gaps in financial protection, as described in the section above.

“Sometimes they (health workers) tell us to buy the drugs ourselves yet …there are times when you don’t have even 10 Kwacha to buy panado, so in situations like these, the patient stays without taking drugs” (FGD12: Female)

“We just advise them to check on the coming week if we do have some drugs” (Medical Assistant, public health centre)

Community residents identified poor access to emergency services as an additional gap in access, and defined it in terms of lack of adequate equipment and staff at public facilities. Specifically, residents complained that compared to CHAM and private facilities, where service provision is generally rated adequate, public facilities lacked basic resources, such as electricity and water, to provide adequate care. They also noted that health staff frequently resided far from the facility, hampering the provision of services in a timely fashion. Health workers confirmed the veracity of community concerns, but attributed shortages in both equipment and staff to circumstances beyond their own control. Staff shortages were cited to explain the public facility overcrowding, resulting in long waiting times.

“Some of the wards have about 60 patients yet they are being manned by two clinical officers maybe with just two nurses, and the health centers that should have at least three or four medical assistants and maybe 8 nurses, they are running on one medical assistant and two nurses, so, we have a serious issue with human resources” (Medical doctor, public hospital).

“Doctors (health workers) stay far from the hospital because there are no hospital houses as such when one falls sick at night and goes to the hospital he cannot be assisted because there is no doctor who works at night” (FGD01: Female).

In addition, both the FGDs and the interviews revealed that accessibility gaps largely result from the uneven geographical distribution of public facilities. FGDs revealed that large portions of the population reside only in the proximity of CHAM/private facilities. If unable to seek care at CHAM facilities due to the affordability concerns described earlier, community residents are forced to travel long distance to receive care free of charge. Respondents reported that due to long distance and lack of adequate transport, they often arrive at public facilities after standard consultation hours and are therefore denied treatment.

“due to long distance which we travel to the hospital(public), we reach the hospital very late as a result we are being chased away by the doctor or being told that the drugs are finished, so most of the times we come back home without getting any health care” (FGD03: Female).

### Acceptability and accommodation related gaps

Community respondents further complained of poor attitudes and behavior on the part of public providers and poor quality of health services. These complaints indicate the existence of additional gaps in access, pertaining specifically to the acceptability and accommodation dimensions, respectively.

Community residents reported rudeness and favoritism as the main negative attitudes of health workers in public facilities, compared to attentiveness and courtesy at private facilities. Respondents described not being listened to and being prescribed the same kinds of drugs, irrespective of their medical condition. Respondents further indicated that these negative attitudes effectively limit access to services, since those community residents who cannot stand the discourteous attitudes of health workers often avoid seeking health care at public facilities.

*“With government hospitals… if you know the doctor or if you are his/her relative, that is when you are given enough medicine, if not that is when you receive just Panado or nothing”* (FGD03: Female)

“At government (health centers), they (health workers) do not listen to our explanations, they give us the prescription form to get medicine at the pharmacy before we finish explaining” (FGD01: Female)

FGD participants in all six FGDs with women further explained that they experience poor attitudes from health workers more often when they are seeking maternal care:

“… *During labor pains and delivery, the nurse used to shout and insult, she used to tell us not to cry because the time we were getting pregnant she wasn’t there…..we are bored and tired of those insults”.* (FGD12: Female)

None of the four public health workers supported the community’s views on their poor attitudes, stating that they provide the best possible care, given the conditions in which they operate. Their colleagues in private facilities, however, fully supported community perceptions and confirmed the existence of important differences in the provider-patient interaction between private and public facilities.

“When a patient comes to a private institution, we give him more time, we listen to his complaints…..because we have time to listen to their complaints as compared to public institutions” (Clinical officer, private clinic)

The cumulative effect of the deficiencies in public service provision is that community residents perceive services provided at the public sector as inappropriate and poor in quality, compared to those provided in the private sector. They, therefore, expressed a stronger preference for seeking care at private facilities.

“We prefer to go to the private hospitals because we get good services. The private doctors are also good and understand our concerns. They know that visiting their facility, it means we are looking for good services” (FGD12: Female)

## Discussion

This study reveals the views and experiences of the residents of rural Malawian communities in regards to the existence of gaps on all three UHC dimensions (population coverage, financial protection and access to services). Its uniqueness lies in its explicit focus on reporting the perspective of community members, voicing the concerns of those rural residents who are rarely given the opportunity to actively contribute towards the health policy debate in their country. Community responses constitute an additional source of evidence to inform current UHC discussions and policy reforms in Malawi, advancing knowledge on gaps in UHC beyond what has already been reported in existing quantitative studies [16,18], expert opinions, and policy analyses [5,7,14,17].

We acknowledge that this study was only conducted in two districts in rural Malawi and among a few purposively sampled respondents, whose views may therefore not necessarily represent the opinions of all community residents and all health workers in Malawi. Due to this limitation, typical of qualitative research, findings from this study cannot be generalized to other populations and contexts, since health system gaps are to a large extent context-specific. However, we trust that lessons from the results are transferable to other rural districts in Malawi where over 80% of Malawians reside [23,37] and where there exist similar health system characteristics [26,30,32,35,39], and at least partially transferable to other rural settings in SSA which experience similar health system characteristics [38].

Our study confirmed the existence of clear interrelated gaps in the three main dimensions of UHC, as defined by rural communities, indicating a synergy between community perspective on UHC and current global debates [4,5,7,40]. In terms of population coverage, the unanimous sense of entitlement to coverage of public funds (tax revenue) at public health facilities expressed by the study respondents, implies that the country has made considerable efforts towards UHC [17]. In practical terms, the existence of geographical inequities in population coverage confirms the assertion that universal health coverage entitlements, as documented on paper and assumed to be offered to the population, often differ substantially in reality [4]. Also, the operational challenges in effectively implementing the SLAs at the local level, as evidenced in our study, supports findings from previous studies on the Malawian SLA [26,36]. This, by implication, suggests a weakness in effectively extending government’s purchasing and regulatory function to the private health sector within a pluralistic health care system like Malawi’s [41].

Furthermore, our findings clearly indicated that geographical disparities in population coverage have resulted in perceived inequities in financial protection. Being located close to or seeking health care from public facilities were perceived to be associated with opportunities for greater financial protection than being located only close to or having to seek health care from private/CHAM facilities. This implies that the provision of the EHP has mostly been effective when considering the financial dimension (i.e. out-of-pocket payments) at public facilities. The existing literature reveals incidences of illegal or informal charges for medical services that ought to be offered free, in some settings [9,42-44]. This evidence has been reported within contexts where direct out-of-pocket payments were previously implemented in the public health sector [9,42-44]. This important financial protection gap was absent in our findings and the findings of earlier published studies within Malawi [26,30,35]. This possibly suggests that informal payments within the public sector are more likely to arise within contexts where free care or exemption systems exist parallel to out-of-pocket payments, rather than in a system like Malawi which has never relied on user fees after independence [45].

Nevertheless, even in the absence of formal and informal payments at public facilities, our findings indicated that communities perceived themselves to be exposed to some financial risk due to out-of-pocket payments for medical treatment rendered at private/CHAM facilities, transportation costs, and purchases of drugs at private pharmacies. The majority of potential financial protection barriers identified in this qualitative study are not likely to be reflected in quantitative cost/expenditure studies. The reason is that rural residents normally perceive such costs as substantially high, unaffordable and potentially catastrophic, and hence, either completely avoid seeking health care or adopt certain coping mechanisms to avoid incurring the cost. Our findings, therefore, support the widely documented evidence confirming such cost avoiding/coping strategies as very important indicators of gaps in financial protection within poor settings [2,35,46]. The literature also acknowledges long distance to health facilities and transportation difficulties as barriers to accessing services that are supposed to be offered for free [10,47,48]. This implies that UHC reforms, including support for community residents to improve access to transport during health care seeking, can facilitate progress towards universal health coverage in rural Malawi, and within other poor SSA settings.

Interestingly, most of the reported gaps in financial protection and population coverage are often triggered by access-related gaps in the public health sector. Affordability of medical costs at private/CHAM facilities and transport costs remain the main access barriers to seeking health care in rural Malawi. In line with earlier studies in Malawi, supply side deficiencies, ranging from drug shortages to perceived poor quality of care, were reported as the main barriers to accessing health care in public facilities [26,30,34-36,39,49]. These perceived access-related gaps, especially supply side deficiencies in availability of medical products, equipment and facilities, are also frequently reported by studies within other SSA settings [50,51]. However, some studies from Burkina Faso, for instance, revealed that, unlike what has been reported in our study, respondents had relatively good perceptions about the attitude of their health care providers [50,51]. This is probably due to contextual differences between the two health systems or to underlying differences in expectations about what constitutes good quality of care. In rural Malawi, these access-related gaps have led to low satisfaction with services provided by public facilities, and hence, a high preference for private/CHAM facilities, as already reported in previous studies [32,35]. This further widens gaps in financial protection, since the private/CHAM facilities collect out-of-pocket payments. It should be noted that although the community perceived better quality of care at private facilities, in line with what was reported in other studies within SSA settings [52,53], the reality of such facilities actually providing high standard quality of care may differ substantially. In rural Malawi, for instance, probably only the CHAM facilities have a better capacity in terms of infrastructure, medical equipment and personnel than most public facilities. The private-for-profit facilities that exist in the study area are mainly individual business organizations, with few staff, who lack the capacity to handle certain serious cases, such as maternal cases. It is not surprising, therefore, that these private-for-profit providers do not qualify for SLAs with the government. The perception of a relatively low quality of care at public facilities, therefore, mainly comes from the increased utilization rates in these facilities, which has been induced by community desire to access health care free of charge. This has led to frequent shortages of medicines and increased providers’ workload, and hence probably less attention spent on clients. Again, this difference in quality of care between public and private health facilities borders on health systems governance, specifically the role and capacity of government to regulate the private health sector.

Several implications for people-centered universal health coverage policy reforms in Malawi, and similar SSA contexts, can be drawn from our study. The clear illustration of an interrelationship of gaps in universal health coverage implies the need for an integrated and inclusive approach to fill existing gaps [12]. To move towards UHC in Malawi, the possibility of an effective public-private partnership needs to be explored, in order to harness the potentials of the private sector to complement the UHC efforts in the public sector [41,54,55]. The contracting arrangement under the SLA in Malawi, therefore, offers great prospects for universal financial protection, if its implementation can be strengthened through governmental commitment to regular payments of bills and expansion to cover all services under the EHP. Other recommendations on how to strengthen the EHP outlined by Chirwa et al. [36] should also be considered. Given that, in Malawi, private/CHAM facilities provide approximately 40% of health services, are perceived to provide the best quality of care, and (especially CHAM facilities) are located mostly in rural areas [23,36], a strategy that completely integrates both the public and private/CHAM sectors will be essential for filling gaps in universal health coverage. UHC can be achieved to the extent that community residents perceive less difference in cost and quality when seeking health care from any type of facility. This could also imply reforms in the purchasing function of the health system, by introducing a third party purchaser, tasked to purchase EHP equitably from both public and private/CHAM facilities [17,56]. This has the potential of reducing geographical inequities in population coverage of public funds, financial protection and access to quality health care [2].

The above recommendation however, needs to be supported by improvement in the quality of services and an expansion of the service provision capacity of the public health sector. However, directly overcoming the access-related gaps in the public health sector is a complex issue, since such gaps are also generally rooted in the low economic development of the districts and of the country [57]. Insufficient funds to supply enough drugs, train more health professionals and adequately motivate them, provide sufficient health facilities, accommodation for health workers and enough ambulances, is one root cause of the supply side gaps [26]. Given the obstacles to raising additional domestic revenue from the traditional UHC revenue sources (taxes and insurance contributions) within poor settings, overcoming these access-related gaps in rural Malawi may require economic empowerment, increased external intervention and alternative innovative mechanisms of raising additional revenue for the health sector [2].

## Conclusions

This study has demonstrated the ability of rural communities to identify and define gaps in universal health coverage through their own experiences and within their own local contexts. From the perspective of rural residents, there exists a unanimous sense of entitlement to coverage of public funds in Malawi. However, uneven distribution of public and private/CHAM facilities, ineffective public-private services level agreements (SLAs), and several shortcomings or gaps in public service provision, have resulted in geographical inequities in effective population coverage, financial protection, and access to quality health services. We recommend that people-centered and health system responsive UHC reforms are needed within Malawi to ensure the simultaneous implementation of appropriate demand and supply side interventions, to tackle the community defined financial protection gaps in the use of private/CHAM facilities and to address several access-related gaps in the public sector. Such reforms need to adopt a bottom-up approach driven by local evidence reflecting context-specific needs. Current UHC reform options being explored within Malawi, such as complementary micro health insurance and performance-based financing, need to target filling the specific universal health coverage gaps identified by community residents. Further research is therefore needed to demonstrate the potential of such reforms to fill context specific gaps.

## Abbreviations

CHAM: Christian Health Association of Malawi; DFG: German Research Society; EHP: Essential health package; FGDs: Focus group discussions; GDP: Gross domestic product; LMICs: Low- and middle-income countries; MASM: Medical Aid Society of Malawi; MFI: Micro Finance Institution; MHI: Micro Health Insurance; MOH: Ministry of Health; NHSRC: National Health Science Research Committee SLA, Services Level Agreement; SSA: Sub-Saharan Africa; USD: United States Dollar; UHC: Universal health coverage; WHO: World Health Organization.

## Competing interests

We declare that we have no competing interests.

## Authors’ contributions

GAA and MDA conceptualized and designed the study and its data collection tools. GBM supported the design of the data collection tools. GAA administered and transcribed the interviews with health care workers, and supervised the data collection. GBM supervised the transcription of the FGDS. All authors participated in the data analysis. GAA wrote the first draft of the manuscript. GBM and MDA revised the draft. All authors read and approved the final manuscript.

## Pre-publication history

The pre-publication history for this paper can be accessed here:

http://www.biomedcentral.com/1472-6963/14/234/prepub

## Supplementary Material

Additional file 1 (electronic): Focus Group Discussion/Interview Guides. Click here for file
